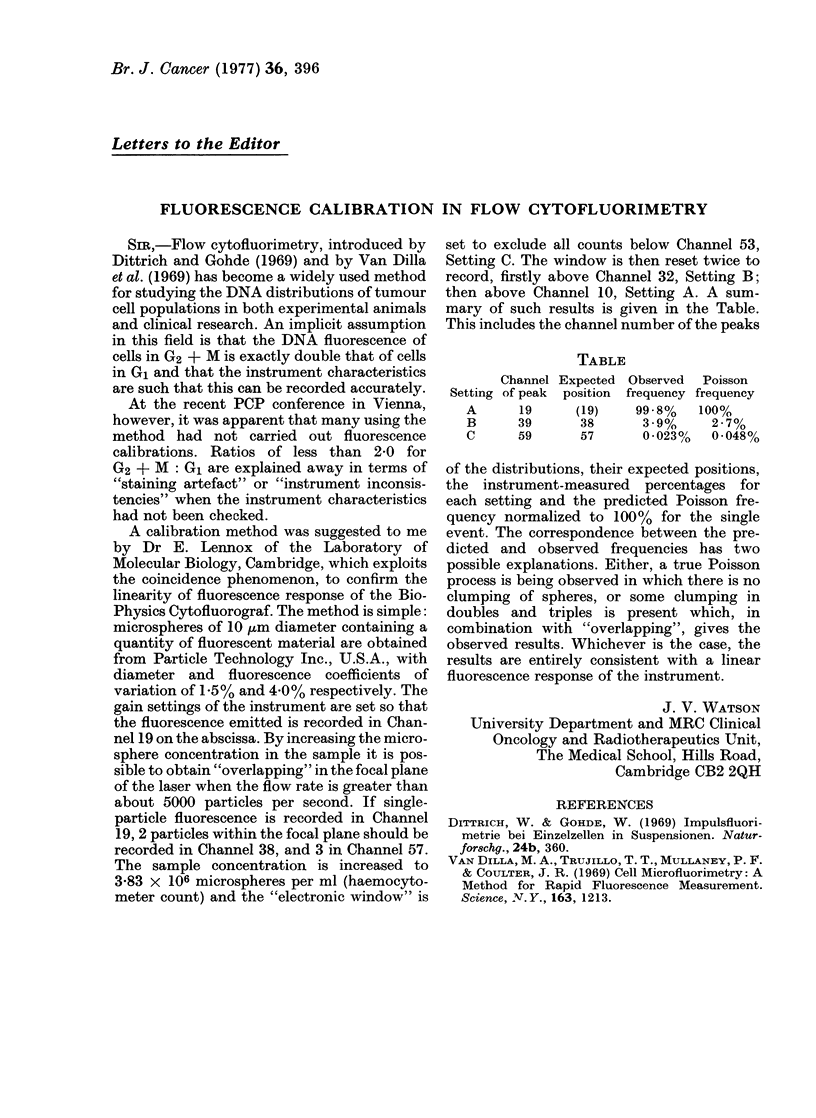# Fluorescence calibration in flow cytofluorimetry.

**DOI:** 10.1038/bjc.1977.206

**Published:** 1977-09

**Authors:** J. V. Watson


*Br. J. Cancer* (1977) **36**, 396

### *Letters to the Editor*

### FLUORESCENCE CALIBRATION IN FLOW CYTOFLUORIMETRY

SIR,—Flow cytofluorimetry, introduced by Dittrich and Gohde (1969) and by Van Dilla *et al.* (1969) has become a widely used method for studying the DNA distributions of tumour cell populations in both experimental animals and clinical research. An implicit assumption in this field is that the DNA fluorescence of cells in $G_2 + M$ is exactly double that of cells in $G_1$ and that the instrument characteristics are such that this can be recorded accurately.

At the recent PCP conference in Vienna, however, it was apparent that many using the method had not carried out fluorescence calibrations. Ratios of less than 2·0 for $G_2 + M : G_1$ are explained away in terms of "staining artefact" or "instrument inconsistencies" when the instrument characteristics had not been checked.

A calibration method was suggested to me by Dr E. Lennox of the Laboratory of Molecular Biology, Cambridge, which exploits the coincidence phenomenon, to confirm the linearity of fluorescence response of the Bio-Physics Cytofluorograf. The method is simple: microspheres of 10 $\mu$m diameter containing a quantity of fluorescent material are obtained from Particle Technology Inc., U.S.A., with diameter and fluorescence coefficients of variation of 1·5% and 4·0% respectively. The gain settings of the instrument are set so that the fluorescence emitted is recorded in Channel 19 on the abscissa. By increasing the microsphere concentration in the sample it is possible to obtain "overlapping" in the focal plane of the laser when the flow rate is greater than about 5000 particles per second. If single-particle fluorescence is recorded in Channel 19, 2 particles within the focal plane should be recorded in Channel 38, and 3 in Channel 57. The sample concentration is increased to $3{\cdot}83 \times 10^6$ microspheres per ml (haemocytometer count) and the "electronic window" is set to exclude all counts below Channel 53, Setting C. The window is then reset twice to record, firstly above Channel 32, Setting B; then above Channel 10, Setting A. A summary of such results is given in the Table. This includes the channel number of the peaks of the distributions, their expected positions, the instrument-measured percentages for each setting and the predicted Poisson frequency normalized to 100% for the single event. The correspondence between the predicted and observed frequencies has two possible explanations. Either, a true Poisson process is being observed in which there is no clumping of spheres, or some clumping in doubles and triples is present which, in combination with "overlapping", gives the observed results. Whichever is the case, the results are entirely consistent with a linear fluorescence response of the instrument.

TABLE

| Setting | Channel of peak | Expected position | Observed frequency | Poisson frequency |
|---|---|---|---|---|
| A | 19 | (19) | 99·8% | 100% |
| B | 39 | 38 | 3·9% | 2·7% |
| C | 59 | 57 | 0·023% | 0·048% |

J. V. WATSON
University Department and MRC Clinical Oncology and Radiotherapeutics Unit,
The Medical School, Hills Road,
Cambridge CB2 2QH

REFERENCES

DITTRICH, W. & GOHDE, W. (1969) Impulsfluorimetrie bei Einzelzellen in Suspensionen. *Naturforschg.*, **24b**, 360.

VAN DILLA, M. A., TRUJILLO, T. T., MULLANEY, P. F. & COULTER, J. R. (1969) Cell Microfluorimetry: A Method for Rapid Fluorescence Measurement. *Science, N.Y.*, **163**, 1213.